# Dr. J. B. S. Holmes

**Published:** 1894-07

**Authors:** 


### DR. J. B. S. HOLMES.

As we go to press we learn that Dr. Holmes has been elected assistant Professor of Obstetrics and Gynecology in the Southern Medical College. Dr. Holmes was formerly from Rome, where he conducted a large sanitarium for the diseases of women. Its destruction by fire more than a year ago induced him to rebuild in Atlanta. His sanitarium here, which is nearing completion, will be a handsome building and well equipped with all the necessaries for good gynecological work. For some time Dr. Holmes has been visiting the hospitals of Philadelphia and New York. He will be an important addition to the Faculty of the Southern Medical College and to the local profession.

